# Anti-Tumor Activity of a Polysaccharide from *Blueberry*

**DOI:** 10.3390/molecules20033841

**Published:** 2015-02-27

**Authors:** Xiyun Sun, Ning Liu, Zhaoxia Wu, Ying Feng, Xianjun Meng

**Affiliations:** 1 College of Food, Shenyang Agricultural University, 120 Dongling Rd, Shenyang 110866, China; E-Mails: sun_xiyun@163.com (X.S.); wuzxsau@163.com (Z.W.); fywjg@sina.com (Y.F.); 2 College of Land and Environment, Shenyang Agricultural University, Shenyang 110866, China; E-Mail: lnbrisk@163.com

**Keywords:** blueberry, a bioactive polysaccharide, anti-tumor, immunoregulation

## Abstract

Blueberries (*Vaccinium* spp.) are rich in bioactive compounds. However, the biological activity of polysaccharides from blueberry has not been reported so far. This study evaluated the anti-tumor and immunological activities of a polysaccharide (BBP3-1) from blueberry in S180-bearing mice. The experimental results indicated that BBP3-1 (100 mg·kg^−1^·d^−1^) inhibited the tumor growth rate by 73.4%. Moreover, this group, compared with the model control, had shown an effect of increasing both the spleen and thymus indices (*p* < 0.05), increasing phagocytosis by macrophages (*p* < 0.05), boosting the proliferation and transformation of lymphocytes (*p* < 0.01), promoting the secretion of TNF-α, IFN-γ, and IL-2 (*p* < 0.05) and improving NK cell activity (*p* < 0.01). From this study, we could easily conclude that BBP3-1 has the ability to inhibit tumor progression and could act as a good immunomodulator.

## 1. Introduction

Blueberry, also known as huckleberry or mustikka, belongs to the *Ericaceae Vaccinium* group of deciduous shrub plants and is popular because of its high health care value, as it is recommended by the FAO as one of the five major healthy fruits [1,2]. At present, the biological activity of blueberry extract is widely studied [3,4,5,6,7]. However, no reports are available describing the effect of soluble polysaccharides, derived from blueberries, on physiological activity.

Recently, many studies have shown that polysaccharides possess antiviral activity, anti-aging properties, resistance to bone marrow suppression, anti-tumor, and immunoregulatory activities [8,9,10] with limited side effects or adverse reactions [11,12], thus making research on polysaccharide immunomodulatory effects popular [13,14,15].

A growing amount of research has shown that polysaccharides possess antitumor activity and in some cases these antitumor effects may be mediated indirectly, possibly through the innate and/or adaptive immune system. Should this be the case there are a number of immune effector functions that polysaccharides have been shown to promote and therefore may be implicated in contributing to the observed antitumor activity, and these include; the enhancement of phagocytosis of macrophage [16], the activation of T and B lymphocytes [11], the activation of natural killers cells (NK cells) and lymphokine activated killer (LAK) cells [17]. In addition, polysaccharides (or peptide-polysaccharides) have been shown to promote the secretion of a range of important immunoregulatory cytokines [18], such as interleukins [19], tumor necrosis factor-α (TNF-α) [20] and interferon-γ (IFN-γ) [16,21]. Hence, finding a new polysaccharide from natural source with anticancer and immunopotentiation activities could be a new tool for cancer therapy [22]. This study aimed to ascertain the anti-tumor activities of a polysaccharide purified from blueberries (BBP3-1) using a sarcoma-180 tumor-bearing mouse model and the possible immune mechanism is discussed.

## 2. Results and Discussion

### 2.1. Purity Determination and Composition Property of BBP3-1

BBP3-1 had no absorption at 280 or 260 nm in the UV spectrum which indicated an absence of protein and nucleic acid. The elution profiles of BBP3-1 from Sephacryl S-300 chromatography showed a single and symmetrical peak as well as the HPLC profile, indicating that it was purified fraction and a homogeneous polysaccharide. The weight-average molecular weight (Mw) of BBP3-1 was 18,643 Da. The constituents of the monosaccharide BBP3-1 were rhamnose, galactose and glucose at a molar ratio of 2:3:4, and it was a β-polysaccharide in which 1-6-glucopyranose was the main chain [23].

### 2.2. Effect of BBP3-1 against the Solid S180 Tumor in Mice

Cyclophosphamide (Cy) is generally used for treatment of various types of cancers and a clinically approved anticancer agent that works by slowing or stopping cell growth [12]. Therefore it was used as a positive control in this experiment.

The size of the tumors in the model control group mice increased significantly, extending to the sternum and clavicle with no clear limits and difficult to remove. The tumor volume in the positive control group and all BBP3-1 treatment groups was much smaller, with clear limits and easier removal. The weights of the excised tumors are shown in Table 1.

**Table 1:** Effects of BBP on S180 tumor growth *in vivo* in mice ($\bar{x}$ ± s, *n* = 10).

Group	Dose mg·kg^−1^·d^−1^	Tumor Weight (g)	Percent Growth Rate Inhibition
Normal control	0	0	—
Model control	0	1.19 ± 0.04	—
Positive control (Cy)	20	0.29 ± 0.02 *	75.6%
High dose	400	0.51 ± 0.03 *	57.3%
Medium dose	200	0.36 ± 0.02 *	70.0%
Low dose	100	0.32 ± 0.02 *	73.4%

Compared with model control, * *p* < 0.05.

As shown in Table 1, BBP3-1 high-dose, medium-dose, and low-dose groups demonstrated anti-tumor effects. The percent growth rate inhibition of S180 tumors were 57.3%, 70.0% and 73.4% respectively. It was interesting to find that the low-dose group caused the greatest inhibition which was similar to that in the Cy-treated group (75.6%).

### 2.3. Effect of BBP3-1 on Immune Organs of Tumor-Bearing Mice

The thymus and spleen are two main immune organs in animals and are the essential links of the defense function as well, the viscera index is a preliminary index of determining the immunity [24,25].

As shown in Table 2, the BBP3-1 medium-dose and low-dose groups showed significant increases compared with the control group in the thymus and spleen indices (*p* < 0.05). Such increases may indicate a positive effect on the immune system and may at least partially explain the antitumor effect. But no significant increase was observed in Cy-treated group.

**Table 2:** Effect of BBP3-1 on the weight of immune organs of mice ($\bar{x}$ ± s, *n* = 10).

Group	Dose mg·kg^−1^·d^−1^	Thymus Index	Spleen Index
Normal control	0	0.68 ± 0.02	4.64 ± 0.16
Model control	0	0.64 ± 0.06	4.39 ± 0.23
Positive control (Cy)	20	0.59 ± 0.09	4.26 ± 0.07
High dose	400	0.70 ± 0.10	4.86 ± 0.13
Medium dose	200	0.77 ± 0.06 *	5.16 ± 0.19 *
Low dose	100	0.77 ± 0.04 *	5.21 ± 0.22 *

Compared with model control, * *p* < 0.05.

### 2.4. Effect of BBP3-1 on Immune Cell Function in Tumor-Bearing Mice

Protective immunity against tumors, in general, is caused by humoral and/or cellular immunity. The humoral defence via antibody response is mediated by B cells and other immune cells involved in antigen processing and presentation. Cell-mediated immune defense is mediated by cytotoxic T cells [19,26].

As shown in Table 3, the effect of Cy (20 mg/kg) markedly increased macrophage phagocytosis (*p* < 0.05) compared to the model group and it could restore macrophage phagocytosis in S180-bearing mice close to that of normal control. The BBP3-1 medium-dose and low-dose groups also showed a significant difference (*p* < 0.05) as compared to the model group, which suggests that BBP3-1 enhanced peritoneal macrophage phagocytosis in the dose range of the present experiment.

**Table 3:** Effect of BBP3-1 on the immune cells function of m ice ($\bar{x}$ ± s, *n* = 10).

Group	Dose mg·kg^−1^·d^−1^	Macrophage Phagocytosis	Lymphocyte Proliferation
Normal control	0	1.96 ± 0.06	0.29 ± 0.00
Model control	0	1.41 ± 0.11	0.21 ± 0.01
Positive control (Cy)	20	1.93 ± 0.27 *	0.35 ± 0.02 **^☆☆^
High dose	400	1.69 ± 0.15	0.23 ± 0.01 *
Medium dose	200	1.84 ± 0.16 *	0.25 ± 0.02 *
Low dose	100	1.81 ± 0.23 *	0.38 ± 0.01 **^☆☆^

(1) Compared with model control, * *p* < 0.05 and ** *p* < 0.01; (2) Compared with normal control; ^☆☆^ *p* < 0.01.

In promoting the spleen lymphocyte proliferation BBP3-1 at the low dose displayed a similar effect to Cy. They also showed significant differences (*p* < 0.01) as compared to the model group and normal group as shown in Table 3. The BBP3-1 medium-dose and low-dose groups also showed a significant difference (*p* < 0.05) as compared to the model group. The results may indicate a role for the immune system in the observed antitumor activity [27]. However, it is also possible that the decreasing the tumor size subsequently resulted in increased or normal levels of proliferation.

Additionally, BBP3-1 *in vitro* had a significant boosting effect on macrophage phagocytosis and lymphocyte proliferation for normal mice, and that effect was dose-dependent at the range of 12.5–100 µg/mL [28]. That was not consistent with the results of this article. This may be due to differences in absorption and metabolism of BBP3-1 *in vitro*.

### 2.5. Effect of BBP3-1 on Immune Factor Secretion in Tumor-Bearing Mice

The cytokines such as TNF-α, IL-2 and IFN-γ were also analyzed in order to obtain additional information on underlying mechanisms by which BBP3-1 might express the anti-tumor effects. In this experiment all the three cytokines monitored exhibited from BBP3-1 dose groups were significantly different in the spleen and serum.

It is well known that TNF-α plays a pivotal role in host defense and can induce the expression of a number of other immunoregulatory and inflammatory mediators to eliminate tumor cells [29]. As shown in Table 4, the BBP3-1 medium-dose and low-dose groups showed increased TNF-α secretion in splenic and serum’s lymphocytes of tumor-bearing mice (*p* < 0.01). The Cy group also showed a strong stimulus (*p* < 0.01). However, the high-dose group had opposing effects on stimulating TNF-α secretion when the spleen and serum were compared (*p* < 0.01). We speculate that low-dose BBP3-1 may indirectly play a role of antitumor activity through the stimulation of release of TNF-α [11].

IL-2 can enhance cytolytic T lymphocytes and the cytolytic activity of NK cells [19]. As shown in Table 5, the concentrations of splenic IL-2 in S180-bearing mice were enhanced by treatment with BBP3-1 and Cy compared to those in the control group (*p* < 0.01). No significant changes were found among the BBP3-1 tested groups. However, all BBP3-1 dose groups and the positive control did not show increased serum IL-2 levels. This characteristic of BBP3-1 was similar to endo-polysaccharide from *Phellinus igniarius* [30] and polysaccharide from *Lentinus edodes* [31].

**Table 4:** Effects of BBP3-1 on immune factors TNF-α in lymphocytes ($\bar{x}$ ± s, *n* = 10).

Group	Dose mg·kg^−1^·d^−1^	TNF-α Concentration ng/L	
		Splenic	Serum
Normal control	0	2.78 ± 0.04	2.69 ± 0.01
Model control	0	2.66 ± 0.04	2.62 ± 0.01
Positive control (Cy)	20	2.93 ± 0.04 **	2.79 ± 0.02 **
High dose	400	3.07 ± 0.04 **∆	2.53 ± 0.02 **
Medium dose	200	3.11 ± 0.05 **∆∆	2.69 ± 0.02 **
Low dose	100	3.15 ± 0.06 **∆∆	2.76 ± 0.02 **

(1) The TNF-α concentration was obtained after the cell culture was diluted 10 fold; (2) Compared with model control, ** *p* < 0.01; (3) Compared with positive control, ∆ *p* < 0.05 and ∆∆ *p* < 0.01.

**Table 5:** Effects of BBP3-1 on immune factors IL-2 in lymphocytes ($\bar{x}$ ± s, *n* = 10).

Group	Dose mg·kg^−1^·d^−1^	IL-2 Concentration ng /L	
		Splenic	Serum
Normal control	0	1.28 ± 0.01	1.31 ± 0.03
Model control	0	1.06 ± 0.01	1.23 ± 0.04
Positive control (Cy)	20	1.25 ± 0.01 **	1.23 ± 0.03
High dose	400	1.17 ± 0.02 **	1.21 ± 0.02
Medium dose	200	1.18 ± 0.03 **	1.26 ± 0.03
Low dose	100	1.19 ± 0.02 **	1.26 ± 0.02

Compared with model control, ** p < 0.01.

IFN-γ can activate macrophages as well as promotes cell-based immune responses. As shown in Table 6, the low-dose group showed the strongest promotion of IFN-γ secretion by splenic lymphocytes and in serum from tumor-bearing mice (*p* < 0.01). However, the high and medium-dose groups showed a negative effect on IFN-γ production by splenic lymphocytes and in serum.

**Table 6:** Effects of BBP3-1 on immune factors IFN-γ in lymphocytes ($\bar{x}$ ± s, *n* = 10).

Group	Dose mg·kg^−1^·d^−1^	IFN-γ Concentration ng/L	
		Splenic	Serum
Normal control	0	1.35 ± 0.04	1.09 ± 0.02
Model control	0	1.32 ± 0.02	1.00 ± 0.02
Positive control (Cy)	20	1.37 ± 0.02 * ↑	1.03± 0.01 *
High dose	400	1.12 ± 0.01 ** ↓	1.63 ± 0.02 **∆∆
Medium dose	200	1.22 ± 0.04 * ↓	1.55 ± 0.02 **∆∆
Low dose	100	1.40 ± 0.03 * ↑	1.53 ± 0.02 **∆∆

(1) Compared with model control, * *p* < 0.05 and ** *p* < 0.01; (2) Compared with positive control, ∆∆ *p* < 0.01; (3) Compared with positive control; ↑ represents a relative increase and ↓ represents a relative decrease compared to the model control.

In summary, the data (as shown in Table 4, Table 5 and Table 6) suggest that BBP3-1 at the low dose could stimulate the secretion of TNF-α, IL-2 and IFN-γ and may therefore promote cell-based immune responses. Further experiments will be required to test this.

### 2.6. Effect of BBP3-1 on Activity of NK Cells from Tumor-Bearing Mice

Natural killer (NK) cells are cytotoxic lymphocytes which play a major role in anti-tumor immunity and can prevent tumor progression and metastasis [32].

As shown in Table 7, each BBP3-1 dose group showed increased NK cell activity (*p* < 0.01). Among the experimental groups, the low-dose group showed the greatest enhancement of NK cell activity. The secretive promotion of TNF-α and IL-2 could enhance the cytolytic activity of NK cells [33] which was consistent with the results of in this paper.

**Table 7:** Effect of BBP3-1 on mouse NK cell activity *in vitro* ($\bar{x}$ ± s, *n* = 10).

Group	Dose/mg·kg^−1^·d^−1^	NK Activity (%)
Normal control	0	32.4 ± 4.0
Model control	0	28.8 ± 3.1
Positive control (Cy)	20	53.1 ± 3.8 **
High dose	400	40.4 ± 1.6 **
Medium dose	200	41.4 ± 3.0 **
Low dose	100	49.1 ± 2.9 **

Compared with model control, ** *p* < 0.01.

Our results above demonstate that BBP3-1 (100 mg·kg^−1^·d^−1^) has significant antitumor activity and that, based on cytokine secretion levels induced by BBP3-1 in serum and spleen, it can be inferred that this polysaccharide may have the potential to stimulate cell-mediated immune responses. Further experiments are required to prove there is a link between the immunostimulatory properties of BBP3-1 and its antitumor activity.

## 3. Experimental Section

### 3.1. Materials

S180 tumor cells were kept in RPMI-1640 with 10% fetal calf serum (FCS) at 37 °C in a 5% CO_2_ atmosphere to sustain its bio-activity.

**Figure 1:**
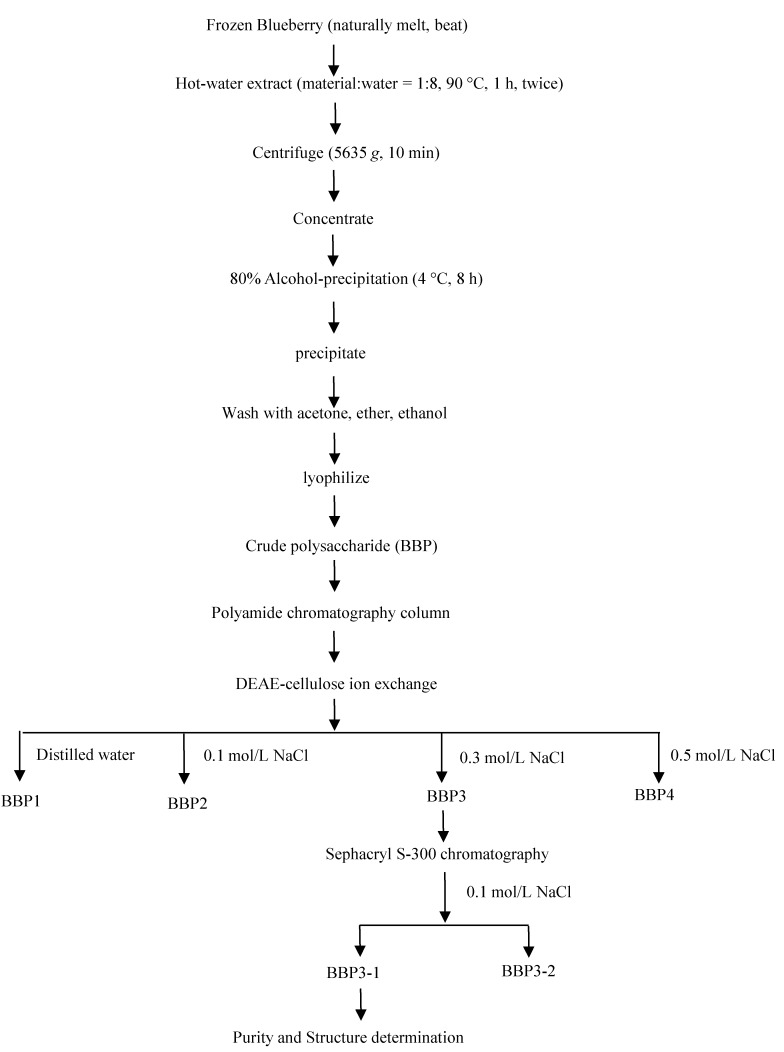
The extract flow diagram of BBP3-1.

The extraction flow diagram of BBP3-1 was presented in Figure 1. A detailed analysis Purity Determination and Composition property of BBP3-1 were previously presented [23].

### 3.2. Reagents

RPMI-1640, DMEM, and DMEM/high glucose (including ketonic acid sodium), penicillin, streptomycin (HYCLONE, Logan, UT, USA), glutamine (AMRESCO, Solon, Tucson, AZ, USA), HEPES, MTT, Trypan Blue (Sigma Co., St. Louis, MO, USA), fetal bovine serum (Hang Zhou Sijiqing Reagent Co., Hangzhou, China), cyclophosphamide (Cy) (Jiang Su Heng Rui Pharmaceutical Co. Ltd., Lianyugang, China), interferon IFN-γ, tumor necrosis factor TNF-α, and interleukin IL-2 ELISA Kit (RND Co., Minneapolis, MN, USA), S180 tumor cells, NK target cell (YAC-1 mice lymphoma cell) (CAS cell bank, Shanghai, China).

### 3.3. Instruments and Equipment

CO_2_ incubator (Thermo Scientific 8000, Vernon Hills, IL, USA), Microplate Reader (BIO-RAD 680, Hercules, CA, USA), inverted microscope (Olympus-CK2, Tokyo, Japan) Precision Electronic Balance (TP-313, Sartorius, Denver, CO, USA), low-speed centrifuge, (SC-2542, Zhengzhou Boke Instrument Equipment Co., Ltd., Zhengzhou, China). 96-well flat culture plate, 12-well flat culture plate, 96-well U-culture plate (Costar, Washington, DC, USA).

### 3.4. Animals

Kun Ming mice (female), aged 4–5 weeks, body weight 20 ± 2 g, were purchased from Chinese Medial Sciences University. The animals were provided with free access to standard rodent chow and water, and were kept at a temperature of 23 ± 2 °C in a 12-h light-dark cycle. The animals were humanely cared for, according to the Standards for Laboratory Animals of China (GB 14923-94, GB 14922-94, and GB/T 14925-94).

### 3.5. Tumor-Bearing Mice Modeling Building

Mice were randomly divided into six groups, including a normal control group, model group, positive control group, BBP3-1 high-dose group, BBP3-1 medium-dose group, and BBP3-1 low-dose group, each group consisting of 10 mice.

S180 tumor cells were diluted to 5 × l0^6^/mL in saline and injected into the mouse left axillary subcutaneous tissue under aseptic conditions, with each mouse receiving 0.2 mL.

After 24 h, the mice from the BBP3-1 dose groups were orally administered with the corresponding dose (respectively, 400 mg/kg, 200 mg/kg, and 100 mg/kg body mass), gavage volume for each mouse is 0.2 mL; the positive control group was administered with cyclophosphamide (20 mg/kg body mass) orally; and the normal control and model groups were administered with distilled water of the same gavage volume (0.2 mL) for 15 days.

### 3.6. Purified Mice Peritoneal Macrophages Cells (PMФ)

24 h after the last drug administration, the mice were sacrificed by cervical dislocation, and blood was taken from the eye and centrifuged (1368 g for 10 min) to obtain serum. The dead mice were then disinfected in ethanol for 5 min.

Under aseptic conditions, the peritoneum was exposed, injected intraperitoneally with RPMI-1640 culture solution without serum, and irrigated adequately (to prevent the viscera from breaking). This was repeated 3–4 times, after which the suspension of peritoneal macrophages (PMФ) was collected, centrifuged (152 g for 5 min) and re-suspended in RPMI-1640 complete culture solution. The fraction of living cells was determined to be >95% by Trypan Blue staining, and the concentration of PMФ cells was adjusted to 5 × 10^6^/mL.

The PMФ cells were cultured in 96-well flat cell culture plates (100 µL per well) in a humidified 5% CO_2_ atmosphere at 37 °C for 2 h. The wells were then washed with RPMI-1640 culture solution, and 100 µL of RPMI-1640 was added. The resulting cells were purified mice PMФ cells.

### 3.7. Index Detection Method

#### 3.7.1. Anti-Tumor Rate, Spleen Index, and Thymus Index

The tumor, spleen, and thymus were removed from the mice. The anti-tumor rate, spleen index, and thymus index were calculated as follows:

$$r_{anti-tumor}=\frac{w_{c}-w_{d}}{w_{c}}\times 100\%$$

where, *w*_c_ is the tumor weight of the model control and *w*_d_ is the tumor weight of the drug-treated animal.

Thymus index = thymus (mg) per body mass (g)

Spleen index = spleen (mg) per body mass (g)

####  3.7.2. Determination of Peritoneal Macrophages Phagocytosis Function

For the neutral red phagocytosis experimental method, purified PMФ cells were centrifuged at 152 g for 10 min after being cultured for 24 h. The suspended cells were then seeded in a 96-well flat cell culture plate (100 µL per well). Added 100 µL of neutral red (1 g/L saline solution) to each well and placed the culture plate inside a 5% CO_2_ incubator for cultivation at 37 °C for 30 min. After that, centrifugation was applied at 152 g for 10 min and the supernatant discarded. The plate was washed with PBS (200 µL per well) three times before adding the lysis agent (100 µL per well) and incubating overnight. The lysis agent was concocted with acetic acid: anhydrous ethanol 1:1 (v/v). 12 h later, an ELISA reader was used to measure the OD value at 540 nm and the strength of PMФ phagocytosis was indicated by the OD_540_ value [33].

#### 3.7.3. Lymphocyte Proliferation Assay

Preparation of cell suspension of spleen lymphocytes from mice:

The aseptically treated spleen of the mice was weighed in RPMI-1640 medium without serum and then rinsed twice. Approximately 5 mL of serum-free RPMI-1640 medium was drawn into a 5 mL syringe, which was then injected into the spleen. The cells in the spleen were then gently washed out. This operation was repeated several times until the outer membrane of the spleen was transparent. The remaining spleen was ground using a syringe and forced through a 100-mesh sieve. All the spleen cell was collected into RPMI-1640 medium and then the cell suspension was prepared.

The cell suspension was then centrifuged at 152 g for 5 min, and the supernatant was discarded. Approximately 3 mL of tris-NH_4_Cl was added to remove red blood cells, followed by centrifugation at 152 g for 5 min. This operation was repeated twice. The cell pellet was then resuspended with serum-free RPMI-1640 medium and centrifuged at 152 g for 5 min.

Resulting cell pellet was then resuspended in complete RPMI-1640 medium and Trypan Blue staining was used to count living cells to confirm >95% viability, and the cell density was adjusted to 5 × 10^6^/mL.

#### 3.7.4. Detection of Spleen Lymphocyte Activity (MTT)

Spleen lymphocytes were added to 96-well cell flat culture plates (200 µL per well). The blank control group (RPMI-1640 complete culture fluid) was set up at the same time, and there were four wells for each group.

The 96-well cell culture plate was cultured for 44 h in a 5% CO_2_ incubator at 37 °C, and then 20 µL of MTT solution was added to each well and cultured for an additional 4 h. The supernatant was discarded after centrifuging at 152 g for 10 min, and 150 µL DMSO was then added to each well. The plate was shaken for 10 min until the purple crystals completely dissolved, and an ELISA reader was used to measure the OD value at 570 nm. The lymphocyte proliferation and conversion rate were reflected by the OD_570_ values [34].

#### 3.7.5. Determination of NK Cell Activity (LDH)

The target cells (Yac-1) were washed with RPMI-1640 medium, viability >95% was confirmed by Trypan Blue staining, and then the cell concentration was adjusted to 1 × 10^5^/mL. Suspended spleen lymphocytes were used as effector cells (E) suspension, and their concentration was adjusted to 5 × 10^6^/mL.

E and T cells (0.1 mL; E/T = 50:1) were added separately into a cell culture plate with U-bottom wells. Each specimen was allocated to four wells. Target cells were included in a natural release well [(Yac-1) and RPMI1640 medium, 0.1 mL each] and a maximum release well [(Yac-1) and 2.5% Triton X, 0.1 mL each], centrifuged at 152 g for 2 min, then incubated in a 5% CO_2_ incubator at 37 °C. After 2 h, the 0.1 mL supernatant from each well was transferred to another culture plate that had been pre-warmed (37 °C), and 0.1 mL of recently prepared LDH substrate solution was added. The reaction was allowed to proceed for 15 min. To stop the enzymatic reaction, 30 µL of 1 mol/L citric acid stop buffer was added. An ELISA reader was used to read the OD_490_ value of each well at 490 nm to calculate the viability of NK cells [35].

$$NKcellviability=\frac{OD_{reaction}-OD_{naturalrelease}}{OD_{\max imumrelease}-OD_{naturalrelease}}\times 100\%$$

####  3.7.6. Detection of IFN-γ, TNF-α, and IL-2

The serum samples and medium supernatants from spleen lymphocytes cultured for 48 h were applied to 3 wells each. The content of IFN-γ, TNF-α, and interleukin IL-2 were examined by ELISA kit. Summary procedures: Preparing reagents, samples and standard → Add prepared sample and standard, incubated 30 min at 37 °C → Plate washed five times, adding HRP-Conjugate Reagent incubated 30 min at 37 °C → Plate washed five times, adding Chromogen Solution A and B incubated 30 min at 37 °C → Add stop solution → Measure within 15 min → Calculation.

### 3.8. Statistical Analysis

All experiments were repeated at least three times. Analysis of variance and *t*-test were used. Data are reported as the mean ± SD (standard deviation) and average. Statistical calculations were performed using SPSS software (version 12.0, SPSS Inc., Chicago, IL, USA).

## 4. Conclusions

This study, to our knowledge, is the first report on anti-cancer and immune adjustment of a polysaccharide (BBP3-1) isolated from blueberry. It is found that the BBP3-1 has the strongly inhibited the growth of S180 tumor and the best dose is 100 mg/kg body mass in this experiment. In addition, the BBP3-1 can up-regulate the immunity of S180 tumor-bearing mice by increasing the thymus index and spleen index, enhancing lymphocyte conversion and multiplication capacity, promoting macrophage phagocytosis, promoting immune factor secretion (TNF-α, IFN-γ, and IL-2), and enhancing NK cell activity. However, elucidation of the detailed mechanism requires further experiments.
